# Supplementary material for: Learning differentially shapes prefrontal and hippocampal activity during classical conditioning
Source: eLife. 2021 Oct 19;10:e65456. doi: 10.7554/eLife.65456 (PMC8545395; doi:10.7554/eLife.65456)
Supplement: Figure 1—source data 1. [file elife-65456-fig1-data1.docx]

Table 1 Neurons recorded per session and mouse in CA1 (green) and PFC (purple)l

| Animals | Pre  Sessions |  | Post  Sessions |  |  |  |  |  | All Sessions |
| --- | --- | --- | --- | --- | --- | --- | --- | --- | --- |
| 1  CA1 AATC | 15 | 14 | 43 | 25 | 64 | 56 | 36 |  | 253 |
| 2 | 40 |  | 85 | 53 | 19 |  |  |  | 197 |
| 3 | 20 | 24 | 54 | 37 |  |  |  |  | 135 |
| 4 | 28 |  | 52 | 39 | 41 | 42 | 42 |  | 244 |
| 5 | 31 | 29 | 34 | 39 |  |  |  |  | 133 |
| 6 | 36 |  | 34 | 33 |  |  |  |  | 103 |
| 7  PFC AATC | 31 |  | 31 | 22 |  |  |  |  | 84 |
| 8 |  |  | 83 | 42 |  |  |  |  | 125 |
| 9 |  |  | 31 | 41 | 38 |  |  |  | 110 |
| 10 | 79 |  | 83 | 80 | 77 | 7 |  |  | 326 |
| 11 |  |  | 62 | 66 | 62 | 65 | 50 | 69 | 374 |
| 12 | 57 |  | 77 | 94 | 48 |  |  |  | 276 |
| 13  CA1-PFC AATC | 68 |  | 34 | 22 | 84 | 41 | 47 |  | 296 |
| 14 | 42  69 |  | 44  22 |  |  |  |  |  | 86  91 |
| 15 | 17  76 |  | 34  49 | 40  22 | 36  64 | 42  16 | 16  66 |  | 185  293 |
| 16 |  |  | 29  14 | 40  61 | 20  35 | 12  52 |  |  | 101  162 |
| 17 | 12  29 |  | 44  16 | 20  32 | 12  43 | 27  44 |  |  | 115  164 |
| Total | 272  378 | 67 | 484  471 | 348  460 | 192  451 | 179  225 | 94  163 | 69 | 1636  2217 |
